# Rhabdomyolysis during high dose interleukin-2 treatment of metastatic melanoma after sequential immunotherapies: a case report

**DOI:** 10.1186/s40425-018-0370-6

**Published:** 2018-06-14

**Authors:** Joseph I. Clark, Shams Bufalino, Shruti Singh, Ewa Borys

**Affiliations:** 1 Cardinal Bernardin Cancer Center, Loyola University Medical Center, 2160 S 1st Avenue, Maywood, IL 60153 USA; 2 Advocate Lutheran General Hospital, Park Ridge, IL USA

**Keywords:** Rhabdomyolysis, IL-2, Metastatic melanoma, Case report

## Abstract

**Background:**

The treatment options for metastatic malignant melanoma have drastically changed recently,including the increased use of immunotherapeutic agents that offer significant responses. Accordingly, it hasbecome common for sequential administration of such agents. Despite this, no guidelines exist on propersequencing or potential unique toxicities associated with such sequencing.

**Case presentation:**

We describe here the first incidence, to our knowledge, of clinically significant rhabdomyolysis associated with high-dose interleukin-2 after prior treatment with ipilimumab, genetically engineered T-cell therapy and subsequent single agent pembrolizumab in a patient with BRAF wild type metastatic malignant melanoma.

**Conclusion:**

Further studies into the biology of sequential immunotherapy in the treatment of cancer are warranted.

## Background

Treatment options for metastatic malignant melanoma have drastically changed over the last decade. Targeted agents, including BRAF and MEK inhibitors, improve progression free survival and overall survival in patients with BRAF-mutated metastatic melanoma but to date durable remissions have not been consistently observed [1–5]. Immunotherapeutic agents including high-dose interleukin-2 (HD IL-2), and checkpoint inhibitors, such as the anti-cytotoxic T-lymphocyte-associated protein 4 (CTLA-4) antibody ipilimumab and the anti-programmed cell death protein 1 (PD-1) antibodies nivolumab and pembrolizumab, have the ability of inducing significant responses [6–15]. Among these treatments however, HD IL-2 has traditionally been the only agent associated with long term, durable remission, lasting > 5 years [7, 16]. Although it is anticipated that such results are likely to be observed with single agent and/or combination checkpoint inhibitors.

An alternative immunotherapeutic option includes talimogene laherparepvec, (T-VEC), an oncolytic virus that mediates local and systemic antitumor activity by direct cancer cell lysis and an “in situ vaccine” effect [17]. Investigational approaches aimed at immune-mediated mechanisms of antitumor activity that remain viable options include dendritic cell vaccines, tumor cell vaccines and engineered T-cells that target tumor cells directly [18–20].

Sequential therapy involving any number of immunotherapeutic agents is not an uncommon approach in the treatment of patients with metastatic malignant melanoma, regardless of BRAF status, once their disease progresses. Formal evaluation of safety and efficacy of such sequential treatments has not been reported. As such, unexpected toxicities are certain to arise. We report here, to our knowledge, the first incidence of clinically significant rhabdomyolysis associated with HD IL-2 after prior treatment with ipilimumab, genetically engineered T-cell therapy and subsequent single agent pembrolizumab in a patient with BRAF wild type metastatic malignant melanoma.

## Case presentation

A 42 year old male presented to his primary care physician with a 20 pound unintentional weight loss over a 3 month period and new left axillary lymphadenopathy. A core biopsy of his axillary mass revealed metastatic malignant melanoma. He had no prior history of a primary melanoma. A staging PET/CT revealed abnormal FDG uptake in his left axilla and small bowel. A left axillary lymph node dissection was performed and revealed 2 of 19 lymph nodes involved with metastatic melanoma, BRAF wild type, the largest of which measured 10.1 cm. His medical history was significant for oligodendroglioma, which was surgically resected eight years prior to presentation, followed by radiation therapy for recurrence five years prior to presentation. He received four doses of systemic ipilimumab for his metastatic melanoma without incident. Post-treatment imaging revealed disease progression with new diffuse subcutaneous, lung, liver and bilateral axillary lymph node metastases.

He subsequently enrolled on a genetically engineered T-cell trial, targeting tyrosinase. He received fludarabine and cyclophosphamide as a conditioning regimen, then his engineered T-cells were infused, followed by one week of low dose IL-2, (72,000 U/kg IV q8 hours). Per the treatment protocol, unfractionated creatinine kinase (CK) levels were obtained just prior to and for two weeks after infusion of the genetically engineered T-cells. The CK levels were within normal limits during the course of this treatment. He initially experienced disease response, however, four months after his T-cell therapy, he again developed diffuse progression with new hilar lymphadenopathy and progression of his lung and axillary lymph node metastases.

He was next treated with three doses of pembrolizumab but post-treatment imaging again revealed disease progression in his lungs. CK levels were not checked during treatment with either ipilumumab or pembrolizumab. Despite multiple lines of therapy, the patient continued to have an excellent performance status, so he thus proceeded to treatment with HD IL-2 (600,000 IU/kg IV over 15 min every 8 h day 1–5 and day 15–19), which began nine months after receiving his engineered T-cell infusion.

During cycle one of course 1 (day 1–5) of HD IL2, he received 10 out of 14 possible doses and experienced the expected adverse effects of hypotension, sinus tachycardia, oliguria, metabolic acidosis, and acute kidney injury. Serum CK was monitored per protocol and was initially normal but peaked at 641 (50–320 IU/L) during the fourth day of treatment without associated symptoms or cardiac findings on EKG.

He had an uncomplicated recovery and was re-admitted to the hospital for cycle 2 of course 1 (day 15–19) of HD IL-2, without complaints and a normal serum CK level of 133 (50–320 IU/L). After 6 doses of HD-IL2, he began to experience diffuse myalgias and rigors. He was noted to have a rapid rise in CK to 2700 and increase in his serum creatinine from 2.5 to 4.4 (0.6–1.4 mg/dL). An EKG revealed sinus tachycardia and his serum troponin level was normal at 0.02 (0.00–0.04 ng/mL). The rise in CK was attributed to rigors and he was continued on therapy. He went on to receive 2 additional doses of HD IL-2. When his CK rose further to 3900 and the myalgias became more severe despite resolution of his rigors, subsequent doses were held.

Further investigation revealed an elevated serum aldolase of 32.7 (1.2–7.6 U/L), and elevated urine myoglobin of 132 (< 28 mcg/L). MB fractionation of CK was not performed. Urinalysis demonstrated large blood without red blood cells. Serial EKGs demonstrated sinus tachycardia but not sequelae of hyperkalemia, such as peaked T waves. Other labs for serologic autoimmunity, e.g. anti-nuclear antibody, anti-double stranded DNA anti-striated muscle antibody and anti-smooth muscle antibody, were not checked. Muscle function was not assessed with electromyography (EMG). He received supportive care and aggressive intravenous hydration with normal saline. He recovered fully from this episode of rhabdomyolysis.

Subsequent staging with a PET/CT revealed a mixed response in his pulmonary nodules with mild improvement in his hilar and axillary lymphadenopathy. Given his full recovery from previous toxicity and the mixed response on imaging, the decision was made to proceed with a second course of HD-IL2 therapy with close monitoring of CK levels and a low threshold for discontinuation of therapy.

He received only two doses of HD IL-2 and again developed diffuse myalgias with a rapid rise in his serum CK level from 184 to 1680. Serum aldolase and urine myoglobin were again significantly elevated, at 16.2 and 3430, respectively. All further doses of HD IL-2 were held and he was again supported with aggressive intravenous hydration. His clinical symptoms resolved and his CK level trended down to the normal range.

**Fig. 1:**
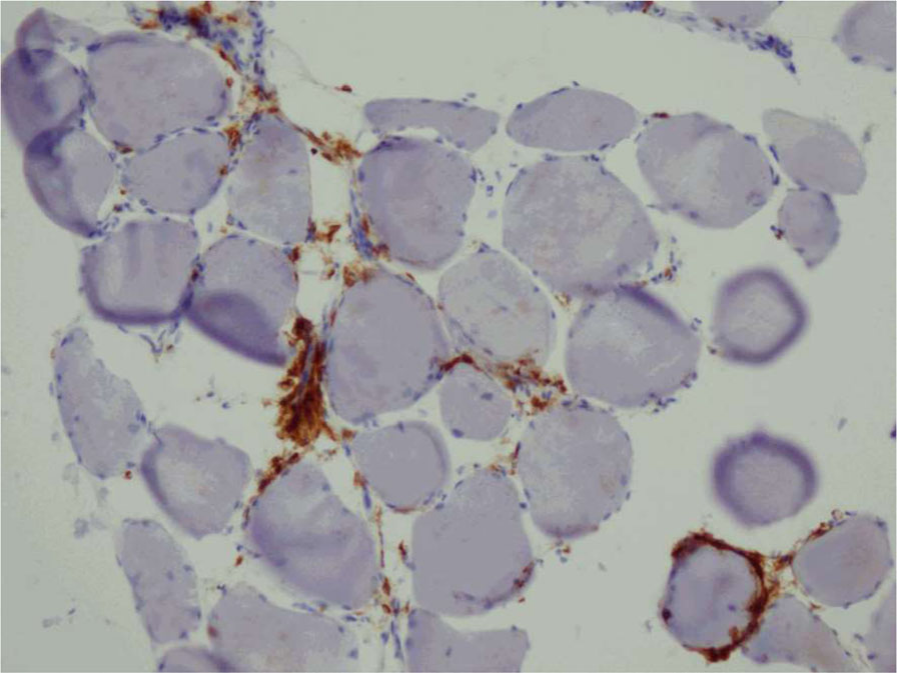
Right thigh muscle biopsy demonstrated scattered necrotic myofibers some of which were infiltrated by macrophages (myophagocytosis). There was scant endomysial inflammatory infiltrate composed of a mixture of CD3 and CD4 positive T-lymphocytes, CD68 positive macrophages and lesser numbers of CD8 positive T-lymphocytes. Type II fiber atrophy was also noted. Findings were those of necrotizing myopathy with accompanying type II fiber atrophy

**Fig. 2:**
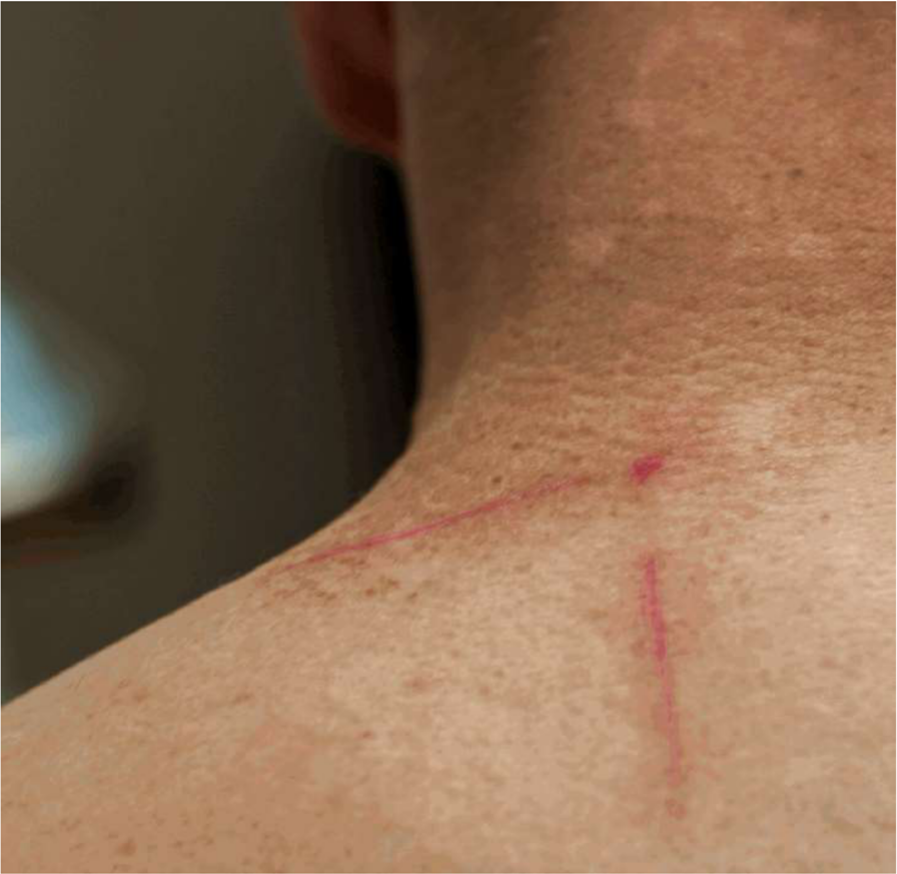
Vitiligo involving neck and upper back

**Fig. 3:**
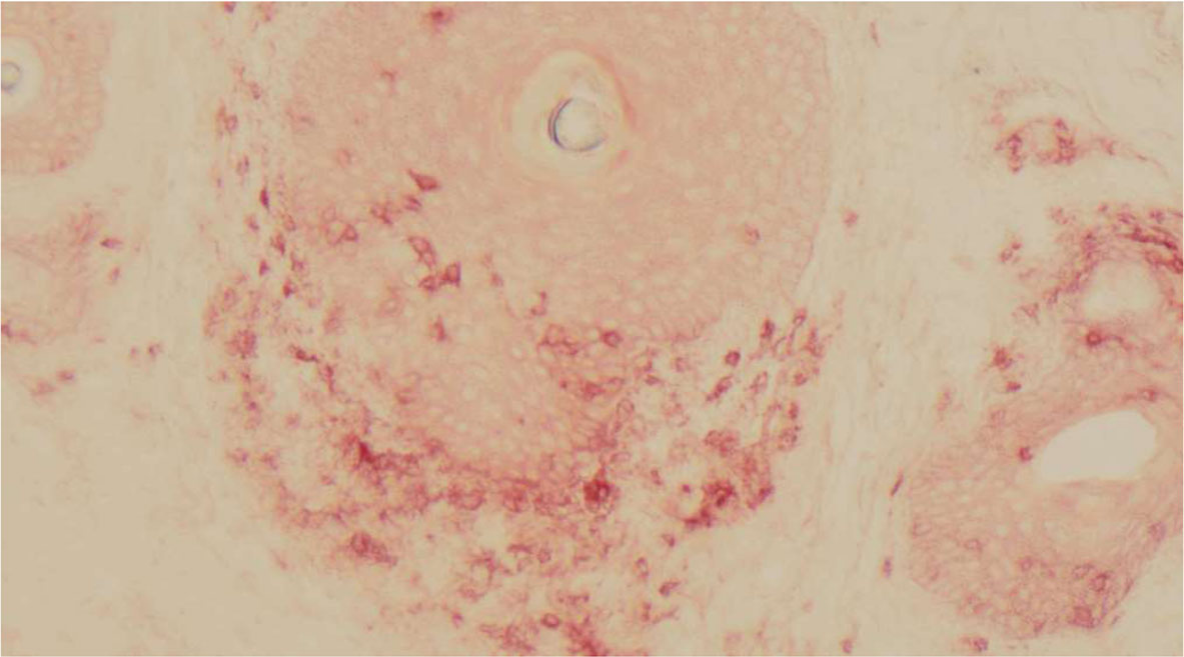
Skin biopsy revealing diffuse CD3+ T-cell infiltration

Due to this unusual toxicity, a muscle biopsy was performed to further evaluate for rhabdomyolysis and to ascertain if his engineered T-cells were present in his muscle tissue. The biopsy revealed rare myofiber necrosis and myophagocytosis and scant endomysial infiltrate. The infiltrate consisted of a mixture of CD3 and CD4 positive T-lymphocytes, CD68 positive macrophages and lesser numbers of CD8 positive T-lymphocytes, suggestive of an immune-mediated toxicity causing necrotizing myopathy (Fig. 1). It was difficult to ascertain if the T-lymphocyte present represented his engineered T-cells. Of note, the engineered T-cells remained detectable in the circulation at this time. He was discharged home and follow up PET/CT imaging revealed a near complete response. At the time of this follow up, he was found to have new onset vitiligo involving his neck, upper back, chest, and upper arms (Fig. 2). A skin biopsy at the edge of his neck vitiligo again revealed CD3+ T-cell infiltration (Fig. 3).

No further HD-IL2 therapy has been administered and he continues to experience a durable response on imaging nearly two and a half years since completion of his HD IL-2 therapy. Of note, CK levels had not been checked when the patient had received prior therapy with ipilumumab or pembrolizumab. CK levels were, however, monitored at the time of treatment with engineered T-cells and were noted to be within the reference range.

## Discussion

To our knowledge, the development of rhabdomyolysis has not been reported as a toxicity associated with HD-IL2 treatment in metastatic melanoma. Prior case reports have cited the development of rhabdomyolysis in the treatment of various disease entities that involved interferon alpha (IFN-α) administration. As early as 1994, Greenfield et al., noted the development of rhabdomyolysis in the treatment of hepatitis C with IFN-α [21]. In 1997, Reinhold et al. described the association of a fatal case of rhabdomyolysis with the use of adjuvant high dose IFN-α for high risk malignant melanoma [22]. An additional case of rhabdomyolysis secondary to adjuvant high dose IFN-α for the treatment of high risk melanoma was detailed by van Londen et al. [23]. In this case it was noted that serum CPK levels remained elevated despite the patient being asymptomatic and IFN-α being held for up to 6 weeks. Anderlini et al. described the case of a female patient who developed rhabdomyolysis after receiving biochemotherapy with IFN-α, IL-2 and chemotherapy (cisplatin, vinblastine and dacarbazine) [24]. This patient’s rhabdomyolysis was believed to be secondary to IFN-α since there were no previously reported toxicities of clinically significant rhabdomyolysis with the other agents.

Interestingly, Esteva-Lorenzo et al. published a case report in 1995 reporting the first case of necrotizing myositis in a patient with metastatic renal cell carcinoma undergoing treatment with HD-IL2 after autologous tumor vaccine [25]. The severe autoimmune mediated muscle injury resolved with discontinuation of therapy. The patient had a disease response to therapy despite this unexpected toxicity.

Immune-related adverse events are known to be associated with immune checkpoint therapy. Bilen et al. described an interesting case of an elderly patient with metastatic papillary urothelial cancer who was treated on a clinical trial with combination ipilumumab and nivolumab and subsequently developed rhabdomyolysis and severe polymyositis. The patient did not have any known prior autoimmune conditions but after his second cycle of treatment presented to the emergency department for lower back pain, muscle weakness and difficulty opening his mouth, eating and speaking. Subsequent laboratory workup was consistent with rhabdomyolyis with polymyositis and the patient received supportive care as well as immunosuppressive treatment with glucocorticoids and infliximab. A paraneoplastic antibody panel was ordered and revealed an elevated anti-smooth muscle antibody titer to 1:61440 (normal 1:≤120). Anti-smooth muscle antibody level on a pre-treatment blood sample was then run and also noted to be elevated to 1:15360 [26]. Similarly, Sakai et al. reported a case of a patient with metastatic melanoma treated with nivolumab who developed severe mononeuropathy multiplex and rhabdomyolysis. This patient did not have a pre-existing history of an underlying autoimmune condition and autoimmune serologies assessed after the onset of the patient’s symptoms were unremarkable [27].

As the literature shows, rhabdomyolysis has not been reported as a toxicity related to HD IL-2 therapy alone. The patient presented here and the patient presented by Esteva-Lorenzo both received genetically modified immune modulating therapy followed by HD IL-2. Notable similarities exist between the two cases including the development of severe autoimmune mediated muscle injury that resolved after discontinuation of HD IL-2 treatment and disease response to therapy. One explanation for this unique toxicity could be that the immune response initiated by either genetically engineered T-cells or tumor vaccine, respectively, was somehow reactivated or enhanced by HD IL-2. In both instances, this manifested as both on-target and off-target responses. An alternative pathophysiologic explanation for the development of rhabdomyolysis is that there was subclinical rhabdomyolysis (or at minimum serum elevation in CK) during treatment with ipilumumab and/or pembrolizumab but that formal diagnosis of rhabdomyolysis went undetected since CK levels are not routinely checked in asymptomatic patients with either of these treatments. Exposure to HD-IL2 therapy subsequently led to an autoimmune reactivation or exacerbation that led to clinically symptomatic rhabdomyolysis.

## Conclusion

The majority of patients with metastatic melanoma will receive multiple lines of various immune mediated therapies during their disease course. It appears that manipulating the immune system at different time points and by various mechanisms may predispose to unexpected immune mediated toxicities and possibly a disease response. The use of tumor vaccine or modified T-cell therapy in combination or sequentially with HD IL-2 may be valuable in the treatment of malignancies such as metastatic melanoma and renal cell carcinoma. Further studies into the biology of sequential immunotherapy in the treatment of cancer are warranted.

## Abbreviations

CTLA4: cytotoxic T-lymphocyte-associated protein 4 (CTLA-4)
HD-IL2: high-dose interleukin-2
PD-1: programmed cell death protein-1
TVEC: talimogene laherparepvec
